# Research advances on artificial intelligence assisted diagnosis and risk assessment in cardiovascular disease using retinal imaging

**DOI:** 10.3389/fcvm.2025.1615857

**Published:** 2025-11-14

**Authors:** Yandan Wang, Weihua Yang, Yan Li

**Affiliations:** 1 Department of Ophthalmology, The Affiliated Traditional Chinese Medicine Hospital, Southwest Medical University, Luzhou, China; 2 Shenzhen Eye Hospital, Shenzhen Eye Medical Center, Southern Medical University, Shenzhen, China

**Keywords:** artificial intelligence, retinal imaging, OCT, OCTA, retina, cardiovascular disease

## Abstract

**Objective:**

Cardiovascular disease (CVD) is the leading cause of death worldwide, and early prediction and prevention are essential to reduce its incidence. In recent years, Artificial Intelligence (AI) techniques have made significant progress in medical imaging analysis, especially in predicting CVD risk from retinal imaging.

**Methods:**

As of August 2025, we searched using several electronic databases including PubMed, Web Of Science Core Collection. Screening was performed based on inclusion and exclusion criteria, and 43 papers were finally selected.

**Results:**

AI shows great potential in predicting CVD risk from retinal imaging [optical coherence tomography (OCT), optical coherence tomography angiography (OCTA), and color fundus photography (CFP)]. Non-invasive eye examinations combined with AI analysis offer the potential for mass screening and early warning.

**Conclusions:**

AI has made significant progress in the field of CVD assisted diagnosis and risk assessment using retinal imaging. Single-modality models have achieved high accuracy, while multimodal models have further enhanced performance. However, challenges remain, including reliance on single-center data and insufficient generalization capabilities. Future steps include building multi-center datasets, developing dynamic risk models, and promoting portable devices for underserved regions. While promising for early CVD prevention, interdisciplinary collaboration is needed to improve generalizability, standardization, and interpretability for higher clinical value.

## 1 Introduction

Cardiovascular disease (CVD) encompasses a range of conditions affecting the heart and blood vessels, including coronary artery disease and peripheral artery disease (PAD) (1). As the most prevalent form of CVD, ischemic heart disease is a leading global cause of disability and mortality and can result in acute myocardial infarction (MI) (2). According to the 2019 Global Burden of Disease report, global CVD-related morbidity and mortality have nearly doubled over the past three decades, with an increasing burden observed among adolescents and young adults (2, 3). This trend underscores the substantial societal and economic impact of CVD (2, 4). Clinical studies indicate that early intervention in high-risk individuals can delay disease progression and improve outcomes. Therefore, early diagnosis, risk prediction, and risk stratification are critical in CVD management. These efforts rely on biomarkers—such as troponin, C-reactive protein, and B-type natriuretic peptide—and functional imaging modalities, including computed tomography and magnetic resonance imaging, which play vital roles in assisting diagnosis, predicting risk, and stratifying patients (5, 6). However, many current CVD assessments are invasive, time-consuming, and primarily geared toward diagnosis rather than prospective risk evaluation. Their results can be influenced by operator experience and clinical expertise (7). Moreover, resource-limited settings often lack the infrastructure for precise diagnosis and effective risk stratification, highlighting the need for more accessible and standardized tools.

Artificial intelligence (AI) has garnered significant interest in healthcare due to its increasing sophistication and expanding applications (8–10). In ophthalmology, AI models have demonstrated the ability to predict systemic disease risks from retinal imaging, enabling risk stratification and opening new avenues for personalized prevention strategies (11). This progress is driven by three key factors: the widespread clinical adoption of high-resolution, non-invasive ophthalmic imaging; the accumulation of large-scale datasets for correlation analysis; and the development of novel analytical methods, including AI (11). Besides, its use in healthcare has great potential to enhance the quality of service to patients (12, 13). Retinal imaging-based AI, in particular, shows broad applicability across various systemic diseases, including endocrine, cardiovascular, neurological, renal, autoimmune, and hematological disorders (14–16). Furthermore, studies indicate that AI can assist in diagnosing CVD such as atrial fibrillation and hypertrophic cardiomyopathy, facilitate disease stratification and phenotyping, and predict clinical outcomes by integrating multimodal medical data (7, 17, 18). Advanced retinal imaging techniques—including optical coherence tomography (OCT), optical coherence tomography angiography (OCTA), and color fundus photography (CFP)—generate high-resolution images. By training on these images, AI can develop powerful predictive models to help clinicians identify at-risk individuals earlier and optimize interventions.

This review examines the latest applications of AI in assisted diagnosing, assessing, and stratifying CVD risk based on retinal imaging. Furthermore, it evaluates the current landscape, including the strengths and limitations of this approach, and discusses future perspectives on using the retina as a window for CVD prediction via AI.

## 2 Method

To ensure the systematicity and reproducibility of this review, the search and selection of literature were conducted following the Preferred Reporting Items for Systematic Reviews and Meta-Analyses (PRISMA) guidelines, with a predefined strategy.

### 2.1 Search strategy

A systematic literature search was performed in August 2025 using the following electronic databases: PubMed and Web of Science Core Collection. The search strategy incorporated Boolean operators (AND, OR) and combined keywords related to artificial intelligence (AI), retinal imaging, and cardiovascular disease (CVD). The search query used in PubMed is provided below as an example: (“artificial intelligence” OR AI OR “deep learning” OR “machine learning”) AND (retina OR fundus OR “retinal imaging”) AND (“cardiovascular disease” OR CVD OR “heart disease” OR “cardiovascular risk”).

### 2.2 Inclusion and exclusion criteria

#### 2.2.1 Inclusion criteria

1. Primary research articles;
2. Human subjects;
3. Focus on AI models applied to retinal imaging (e.g., CFP, OCT/OCTA) for CVD prediction, risk stratification, or clinical assistance;
4. Full text available in English;
5. Publication date between January 1, 2018, and August 1, 2025.

#### 2.2.2 Exclusion criteria

1. Animal studies;
2. Reviews, commentaries, conference abstracts, or book chapters;
3. Studies not focused on CVD (e.g., diabetic retinopathy only) or not employing AI models;
4. Articles for which full text was unavailable.

### 2.3 Study selection process

All identified records were imported into EndNote for duplicate removal. The screening process consisted of two phases: Initial Screening: Two investigators independently reviewed the titles and abstracts of all retrieved articles to exclude clearly irrelevant studies. Full-Text Review: The full texts of potentially eligible articles were obtained and assessed independently by the same two investigators against the inclusion and exclusion criteria. Any disagreements were resolved through discussion or, when necessary, by a third reviewer. Additionally, the reference lists of included articles were manually screened to identify any additional relevant publications.

### 2.4 Results of study selection

**Figure 1:**
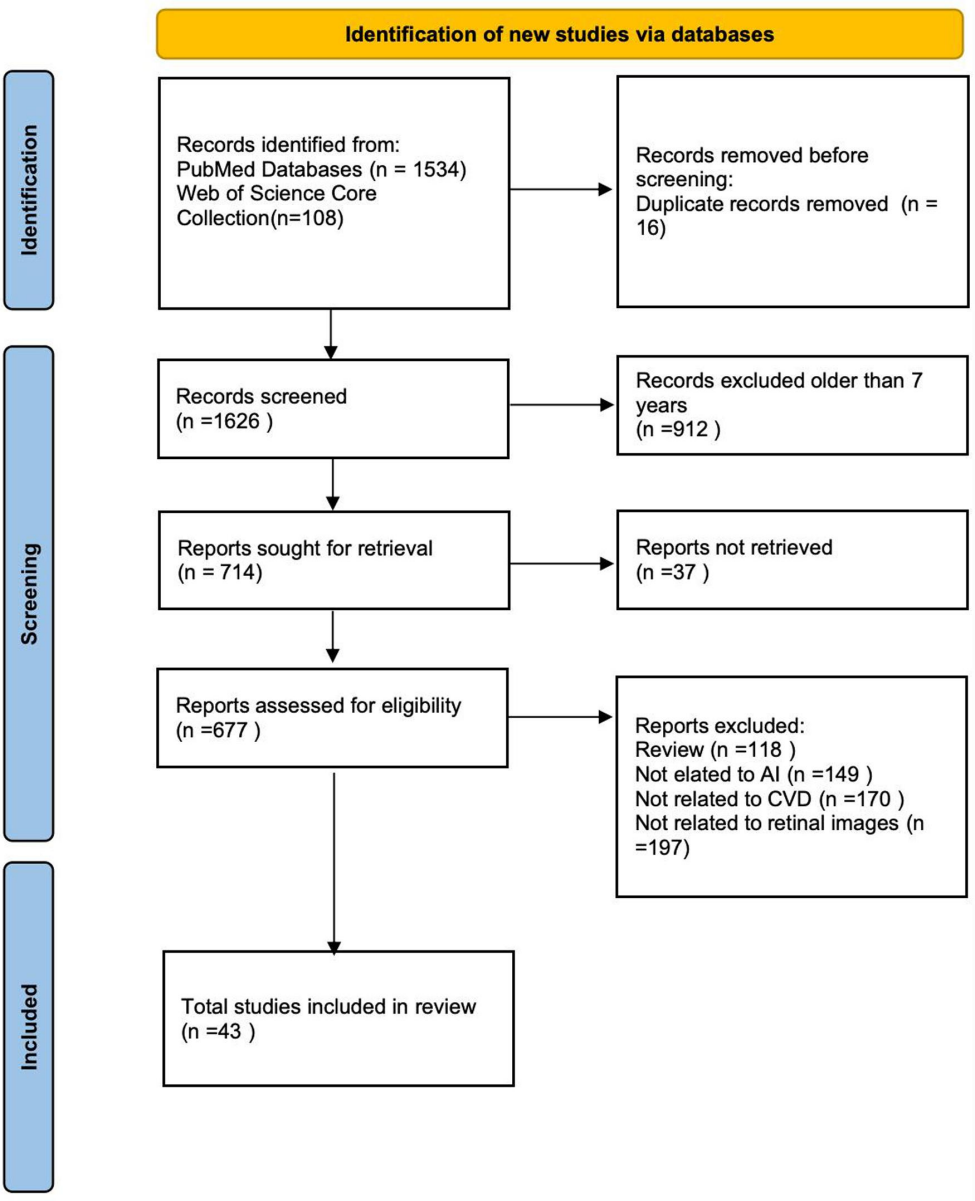
The literature screening process of this review.

The initial database search yielded 1,642 records. After removing 16 duplicates, 912 records were excluded based on publication year, and 37 were excluded due to unavailability of full text. Following the application of exclusion criteria, 634 articles were further excluded. Ultimately, 43 studies met all eligibility criteria and were included in this review. A detailed flowchart of the study selection process is provided in accordance with the PRISMA guidelines (Figure 1).

## 3 Current status of research on AI for assisted diagnosis, prediction, and stratification of CVD risk using retinal imaging

Deep learning (DL), a key branch of AI, is extensively employed in medicine to analyze complex biomedical data (19). AI algorithms used in healthcare, primarily encompassing machine learning (ML) and DL, are designed not only to process large-scale datasets but also to assist clinicians in identifying and monitoring disease risk (20–22). At the same time, a substantial number of studies have proposed the implementation of AI for the domain of CVD imaging (23, 24). The retina provides a unique window for non-invasive assessment of the human microvasculature. High-definition ophthalmic imaging techniques—such as OCT, OCTA, and CFP—allow detailed visualization of retinal architecture. Consequently, AI-driven analysis of retinal imaging has emerged as a promising tool for predicting CVD risk. Evidence indicates that such analysis can identify CVD risk factors, predict clinical events, and detect associated biomarkers (25). For instance, AI models can estimate CVD parameters with accuracy comparable to expert graders, demonstrating significant associations between retinal vessel caliber and risk factors like hypertension, body mass index, and cholesterol levels (26). Retinal vascular measurements have not only been correlated with these risk factors but also linked to incident CVD events (26). A study in Kenya highlighted that while the accuracy of ML-based parameter estimation was slightly lower than in trained reference populations, it represents a critical step toward accessible, early CVD screening in resource-limited settings (27). This approach thus provides a non-invasive and convenient method for risk assessment by leveraging microvascular information from retinal imaging. However, the field remains in its early stages, facing challenges such as limited dataset size and insufficient model generalizability.

AI models have shown promise in assisted diagnosing and predicting risks for specific CVDs using retinal imaging, including MI, hypertensive heart disease, carotid atherosclerosis, heart failure, coronary heart disease, and PAD, as summarized.

### 3.1 AI use CFP to assist in the diagnosis and prediction of CVD and to stratify CVD risk

AI models assist in diagnosing CVD, predicting patient risk, and stratifying patients by analyzing CFP to identify abnormal retinal features, such as variations in vascular caliber and morphology. The predictive accuracy of these models, as measured by area under the curve (AUC), typically exceeds 0.9, with some outperforming or usefully complementing traditional risk scores. For instance, a hybrid Inception V3-VGG16 model has demonstrated a high accuracy of 99.5% in predicting CVD risk from CFP. Nevertheless, most developments stem from single-center studies, limiting their generalizability. Furthermore, while some models offer a degree of interpretability, the decision-making mechanisms of many remain opaque. Future research should therefore prioritize multi-center external validation and clinical translation to enhance practical utility and robustness, ultimately facilitating their deployment in resource-limited settings. Detailed characteristics of the supporting studies are summarized in Table 1.

**Table 1:** AI use CFP to assist in the diagnosis of CVD, predict CVD risk, and stratify patients.

Al algorithm	Country	Dataset	Year	Device used	Disease type	Imaging type	Model performance
Inception-v3 (28)	Korea	20,130 individuals	2020	Canon CR6-45NM, Kowa nonmyd7	Predict CACS	CFP	The model AUROC reached 83.2%, outperforming single clinical parameters.
Inception-ResNet-V2 (29)	China	412,827 individuals	2022	Not detailed	Predict ICVD 10-year risk	CFP	This model predicts the 10-year risk of ICVD with an AUC of 0.85–0.971.
Reti-aging score (30)	China	4,376 individuals	2024	Canon CR-2 Digital Retinal Camera, Canon CR-2 AF Digital Retinal Camera	Predict CVD risk	CFP	This model can efficiently predict vascular aging and CVD risk through CFP, with an AUC of 0.779–0.826. It outperforms single clinical parameters (such as age, hypertension, etc.) and specialist physicians.
FCHOA-based DNFN (31)	Greece	1,000 images	2022	Nidek AFC-210 fundus camera	Predict CVD risk	CFP	The model predicted CVD risk with an accuracy rate of 91.6%, a sensitivity of 92.3%, and a specificity of 91.9%, which was superior to traditional models.
SIVA-DLS (26)	Singapore, New Zealand, Australia, China, Korea, UK	70,000 images	2020	Canon CR-DGi 10D, Canon CR6-45NM, Topcon 3D OCT-1000 Mark II	Predict CVD risk	CFP	The model's ability to predict CVD risk had an intraclass correlation coefficient of 0.82–0.95 with expert scores.
EfficitNetB0 (32)	India	6,026 individuals	2024	Snethra Classic HD	Predict CVD risk	CFP	The model's accuracy in predicting CVD risk is 87.5%.
CNNs based on transfer learning (33)	Many country	210,494 images	2024	Not detailed	Predict CVD risk	CFP	The model predicted CVD risk with an accuracy rate of 92.1%.
FCBOA-SpinalNet (34)	China	1,000 images	2024	Not detailed	Predict CVD risk	CFP	The model achieved a CVD risk prediction accuracy of 0.913, significantly outperforming traditional models.
DenSFFNet (35)	Many country	1,860 images	2025	Not detailed	Predict CVD risk	CFP	The model achieved an accuracy rate of over 90% in predicting CVD risk in a multicenter dataset.
Hybrid Inception V3-VGG16 (36)	China	1,433 images	2024	Not detailed	Predict CVD risk	CFP	The model predicts CVD risk with an accuracy rate of up to 99.5%.
rpCVD (37)	Australia	27,595 individuals	2025	Mediworks FC162	Predict 10-year CVD risk	CFP	The AUC of the model for predicting 10-year CVD risk was 0.672, which was comparable to the WHO CVD risk score (AUC = 0.693).
An ordinal regression DL model (38)	UK, Australia	35,053 individuals	2025	Topcon 3D OCT 1000 Mark II, Topcon TRCNWS, Mediworks FC-162	Predict 10-year CVD risk	CFP	The model predicts 10-year CVD risk and stratifies it using CFP, solving the problem of underestimation of retinal scores.
RetiCAC (39)	Korea, Singapore, UK	216,152 images	2021	Not detailed	Predict CVD risk and stratification	CFP	The model significantly improved the predictive ability of the pooled cohort equation (PCE) for moderate-risk and marginal-risk groups. As a complementary tool to PCE, it optimized decision-making for moderate-risk groups (e.g., 7.5%-20% 10-year risk).
Reti-CVD (40)	UK	48,260 individuals	2023	Topcon 3D OCT-1000 Mark II, AFP-210, TRC-NW8, Nonmyd A-D	Refine CVD risk stratification	CFP	For individuals with QRISK3 Cardiovascular Risk Algorithm scores in the borderline risk range (7.5%–10%), further stratification analysis can be performed.
DL-FAS (41)	Korea	37,523 individuals	2020	Canon CR-2	Predict carotid atherosclerosis	CFP	The model predicted an AUROC of 0.713 for carotid atherosclerosis, with an accuracy of 0.583 and a sensitivity of 0.891.
RetiCAC (39)	Korea, Singapore, UK	216,152 images	2021	Not detailed	Predict CAC	CFP	The model predicted an AUC of 0.742 for CAC, which was superior to single clinical parameters such as age and blood glucose levels.
Attention-based Multiple Instance Learning (42)	Germany	135 individuals	2022	EIDON Wide Field True Color Confocal Fundus Imaging System	Diagnosis of PAD	CFP	The AUC of this model for diagnosing PAD is 0.89.

#### 3.1.1 AI predicts CVD risk using CFP

Recent advances in AI have significantly improved CVD risk prediction using CFP. Evidence indicates that CFP captures biomarkers predictive of future CVD risk, with DL-derived risk scores independently associated with CVD events (43–45). Retinal features—including ischemic perivascular lesions, subretinal drusen-like deposits, and microvascular metrics such as vessel density, caliber, tortuosity, and fractal dimension—have been established as clinically useful indicators for systemic disease assessment (46, 47). For example, a U-Net-based DL model demonstrated a significant association between retinal microvascular density and fractal dimensions with congestive heart failure, reinforcing the retina's role as a window into cardiovascular health (47).

Several studies have developed specific DL models for CVD prediction. A model based on the Inception-v3 architecture predicted high coronary artery calcium scores (CACS >100) from CFP using vascular and macular features, achieving an area under the receiver operating characteristic curve (AUROC) of 83.2%—outperforming single clinical parameters and comparable to age-based indicators (28). Another model developed to estimate 10-year ischemic cardiovascular disease (ICVD) risk showed strong performance (AUC 0.85–0.97) and was trained on a large dataset of nearly 400,000 participants, enhancing its generalizability (29). Wang et al. introduced a DL-based retinal aging score (Reti-aging score) that efficiently predicts vascular aging and CVD risk from CFP, with an AUC of 0.779–0.826, outperforming both single clinical parameters (e.g., age, hypertension) and specialist physicians (30). The same model also predicted new-onset hypertension and carotid artery plaque with AUC values of 0.703 and 0.705, respectively (30).

Several advanced AI models have been developed to predict hypertension-related CVD risk using retinal imaging. Srilakshmi et al. introduced a deep neuro-fuzzy network combined with a fractional calculus-based optimization algorithm, achieving an accuracy of 91.6%, sensitivity of 92.3%, and specificity of 91.9%, outperforming conventional models (31). Similarly, the Singapore I Vessel Assessment-Deep Learning System (SIVA-DLS) automates retinal vessel diameter measurement, showing high agreement with expert graders (intraclass correlation coefficient: 0.82–0.95) (26). Other approaches include a hybrid computer vision and DL model with 87.5% accuracy (32), a convolutional neural network that integrates multi-feature fusion to capture microvascular abnormalities (33), and an osprey gannet optimization-based model reaching 92.1% accuracy (33). Additional high-performing frameworks include a fractional chef-based optimization algorithm combined with SpinalNet (accuracy: 91.3%) (34), a Dense Spiking Forward Fractional Network exceeding 90% accuracy in multi-center datasets (35), and a hybrid Inception V3-VGG16 model achieving 99.5% accuracy by leveraging both global and fine-grained retinal features (36). The latter also offers rapid processing, making it suitable for elderly screening in resource-limited settings (36).

In summary, most CFP-based AI models demonstrate high predictive accuracy (>90%), often with screening times under one minute. Integration of retinal microvascular features with clinical data further improves performance. These non-invasive, cost-effective tools show particular promise for early CVD risk screening in underserved populations. Nevertheless, many models rely on single-center, limited-sample datasets, constraining generalizability, and further validation in diverse cohorts is needed before broad clinical adoption.

#### 3.1.2 AI stratifies CVD risk using CFP

CVD risk stratification is crucial for the prevention and management of CVD (48). AI models using CFP can not only predict CVD risk, but also stratify CVD risk. A study found that only 25.8% of participants had undergone CVD risk assessment (37). This method not only identifies high-risk and borderline-risk groups but also facilitates more precise early intervention, thereby improving patient survival rates. Retinal vessels, due to their anatomical specificity, have emerged as a potential tool for CVD risk stratification (48). Consequently, an increasing number of research and development efforts are focused on developing AI models for CVD risk stratification using CFP. Research has found that AI models based on CFP [Residual Neural Network 18 (ResNet 18)/Multiple Instance Learning] can be used for screening and grading diabetic cardiac autonomic neuropathy (49). Several AI models demonstrate the potential of CFP for CVD risk stratification. One rpCVD model achieved an AUC of 0.672 in predicting 10-year CVD risk, performing comparably to the World Health Organization (WHO) risk score (AUC = 0.693) (37). Risk classifications (low/moderate/high) were consistent with the WHO score in 63.4% of participants, and both patients (92.5%) and general practitioners (87.5%) reported high satisfaction with the tool (37). This model can be incorporated into primary care workflows to help address the limited coverage of conventional risk assessment methods (37). An ordered regression DL model has also been developed to predict and stratify 10-year CVD risk based on CFP (38). By integrating significance analysis and imaging decomposition techniques—such as vessel or macula removal experiments—the model provides interpretable predictions by identifying key retinal features influencing risk stratification (38). The Retinal Imaging-Based CVD (Reti-CVD) tool identified a 10-year CVD risk of 13.1% in its high-risk group (40). Compared to QRISK3, the current UK standard, Reti-CVD improved stratification of borderline-risk individuals and detected high-risk patients missed by QRISK3 (e.g., 8.6% of the high-risk group had a QRISK3 score of only 0%–5%) (40). These findings support its use as a non-invasive aid for clinical decision-making, such as initiating statin therapy (40).

In summary, AI-enhanced retinal imaging allows more precise and comprehensive CVD risk stratification, which may facilitate personalized early intervention and slow disease progression. However, few CFP-based stratification models currently exist, most are derived from single-center studies, and clinical practicality remains limited.

#### 3.1.3 AI assisted diagnoses CVD using CFP

In addition to risk prediction, AI has also demonstrated diagnostic potential for specific CVD subtypes. The DL-funduscopic atherosclerosis score (DL-FAS) model enhances the predictive capability of cardiovascular mortality risk based on the Framingham Risk Score (FRS), demonstrating significant value particularly in risk stratification among individuals with moderate risk (FRS 10%–19%) (41). This model holds promise as an adjunct tool for CVD risk stratification (41). Additionally, by quantifying atherosclerotic features in carotid angiography, the model achieved an AUROC of 0.713 for predicting carotid atherosclerosis, along with an accuracy of 0.583 and a sensitivity of 0.891 (41). A multicenter, large-scale study found that retinal imaging of coronary artery calcium (RetiCAC) is comparable to computed tomography-measured coronary artery calcium (CAC) in terms of risk stratification performance (39). Furthermore, it significantly improves the predictive ability of the pooled cohort equation (PCE) for intermediate-risk and borderline-risk groups, serving as a complementary tool to PCE to optimize decision-making for intermediate-risk groups (e.g., 7.5%–20% 10-year risk) (39). Besides, this model predicts CAC achieved an AUC of 0.742, outperforming single clinical parameters such as age and blood glucose levels (39). This aids in the early assisted diagnosis and management of CAC. Furthermore, an attention-based multi-instance learning model has been validated for its feasibility in the early assisted diagnosis of PAD (42). By leveraging microvascular changes in the optic disc and temporal vascular arcade, this model offers a novel perspective for the systematic assessment of atherosclerosis (42). Currently, there is limited literature on the assisted diagnosis of CVD using CFP, and it lacks clinical practicality. In the future, more attempts can be made to develop models for diagnosing CVD by combining clinical data, population information, and other factors.

### 3.2 AI predicts and stratifies CVD risk using OCT/OCTA

AI models utilizing OCT and OCTA can identify vascular and retinal abnormalities to predict CVD risk and stratify patients. These models generally demonstrate high predictive accuracy, with some outperforming or usefully complementing conventional risk scores. Approaches such as extreme gradient boosting (XGBoost), convolutional neural networks (CNN), and anatomy-sensitive inference networks (ASI-Net) have further improved the accuracy of automated analysis and prediction. However, challenges related to data standardization, clinical translatability, and the limited number of available models remain. Future work should prioritize the integration of multimodal data—combining OCT, OCTA, and clinical information—as well as prospective validation in cohort studies to support the translation of these technologies into clinical practice. A detailed summary of the included studies is provided in Table 2.

**Table 2:** AI predicts and stratifies CVD risk using OCT/OCTA.

Al algorithm	Country	Dataset	Year	Device used	Disease type	Imaging type	Model performance
ML models and DL models (EfficientNetV2-B3) (50)	France	491 individuals	2024	PLEX Elite 9000	Predict Neurocardiovascular Risk	OCTA	ML Model: SVM achieved the best performance with an AUC of 0.98 and an accuracy of 85%. DL Model: EfficientNetV2-B3 demonstrated a balanced accuracy of 68%.
Supervised ML model (51)	France	144 individuals	2021	TRC NW68, CIRRUS HD-OCT	Predict CVD risk	CFP, OCTA	The model's accuracy in predicting CVD risk ranges from 75.64% to 96.53%.
RET Found (52)	UK, China, France, Spain, South Korea, India	1,640,612 images	2023	Topcon 3DOCT-2000SA, Canon CR1/OGI/CR2, Topcon NW, SPECTRALIS	PredictMI and heart failure	CFP, OCT	The model predicted AUROC values of 0.737 and 0.794 for MI and heart failure, respectively.
CNN and Mobile Network Version 2 (53)	Australia	247 individuals	2023	Carl Zeiss CIRRUS HD-OCT Model 5000	Predict congestive heart failure risk	OCTA	The AUC for predicting congestive heart failure risk was 0.61.
k-Nearest Neighbors, Naive Bayes Classifier (54)	France, Luxembourg	120 individuals	2025	Not Detailed	Predict coronary artery disease risk	OCTA	The accuracy of the model in predicting coronary artery disease risk (up to 86%) is significantly higher than that of traditional logistic regression models (78.7%).
ML model (55)	UK	2,824 individuals	2025	Topcon 3D OCT 1000 Mk II	Classification of heart failure subtypes	OCT	The ML model analyzes retinal OCT features and achieves the best-performing non-invasive classification of heart failure subtypes (AUC 0.70) for the first time.
Cardiac Risk Assessment DL Model (56)	UK	33,370 individuals	2024	Topcon 3D OCT 1000 Mark II	CVD risk stratification	OCT	The model can accurately distinguish between high-risk and low-risk individuals, consistent with known CVD risk trends.

#### 3.2.1 AI predicts CVD risk using OCT/OCTA

OCT is a high-resolution imaging technique capable of capturing the layered structure of the retina. Studies have shown that changes in retinal thickness and structure are associated with CVD. The risk monitoring and healthcare assistance system model identified certain retinal fundus features in OCT retinal imaging that were significantly associated with the development of aortic aneurysms and adverse aortic events (57). This suggests that OCT may be a promising tool for the early detection and intervention of aortic aneurysms and adverse aortic events (57). A model based on interactive ML technology has, for the first time, enabled automated detection of reticular intraretinal perivascular lesions (RIPLs) and subretinal drusenoid deposits (SDDs) using a small-scale OCT dataset (58). Its efficiency (6-hour training) and high specificity (>90%) provide a new tool for studying retinal biomarkers of CVD (58). OCTA is a high-resolution vascular imaging technique. By enhancing the visualization of deep capillary networks and the choroid, it facilitates the identification of different types of retinal capillary networks and provides detailed imaging of retinal microvascular structures (59–63). OCTA can detect early changes in retinal microvasculature, which may be early biomarkers of CVD (50, 51, 53, 64). A prospective cross-sectional study found that the density of the superficial retinal capillary plexus measured by OCT-A correlates with CVD risk profiles and impaired left ventricular ejection fraction in patients with high-risk CVD status (65). Therefore, quantitative retinal microvascular data can be considered a valid surrogate for CVD risk profiles and may enhance CVD risk assessment. This represents the first evidence linking OCT-A metrics to CVD risk (65). Studies have shown a significant correlation between retinal capillary density and markers of adverse cardiac remodeling, providing a new perspective for predicting the risk of systemic diseases such as CVD (66). A prospective observational cohort study demonstrated that preoperative retinal hypoperfusion independently predicted an elevated risk of perioperative adverse cardiovascular events in patients with coronary heart disease, highlighting retinal microcirculation assessment as a non-invasive biomarker to inform cardiac surgical prognostication and guide personalized therapeutic strategies (67). Supervised ML models combining CFP and OCTA data demonstrated high accuracy (up to 96.53%) in predicting CVD risk (51). The retinal foundation model (RETFound) model achieved AUROC values of 0.737 and 0.794 when using CFP and OCT imaging to predict MI and heart failure, respectively (52).

The AUC of the CNN model for predicting congestive heart failure risk under 3 × 3 mm high-resolution OCTA imaging scanning was 0.61 (53). The scanning performance significantly decreased under 6 × 6 mm resolution OCTA images and 8 × 8 mm resolution OCTA images (AUC ≤ 0.5) (53). Based on retinal OCT-A patient images from the open-source RASTA dataset, a study evaluated the accuracy of ML and DL algorithms in predicting CHA₂DS₂-VASC neurocardiovascular risk scores (50). The findings revealed that EfficientNetV2-B3 is a suitable DL model for retinal OCT-A imaging, correctly predicting risk in 68% of cases (50). This contributes to the assessment of future neurocardiovascular characteristics (50). A study combining traditional cardiovascular risk factors employed multiple ML algorithms (such as k-nearest neighbors, naive bayes classifier, support vector machines, etc.) to predict the risk of coronary artery disease. The classification accuracy of the ML algorithms (up to 86%) was significantly higher than that of traditional logistic regression models (78.7%) (54). As a non-invasive diagnostic method, OCTA holds promise as a new biomarker for CVD (54).

These findings underscore the potential of OCT and OCTA as powerful, non-invasive tools for detecting early retinal microstructural and microvascular changes linked to CVD, offering insights beyond traditional risk assessment methods. The integration of AI-driven analysis with high-resolution retinal imaging enhances predictive accuracy, enabling more precise CVD risk stratification and early intervention strategies. Future research should focus on standardizing imaging protocols and expanding multicenter datasets to further validate these innovative biomarkers for clinical adoption.

#### 3.2.2 AI assisted diagnose CVD and stratify risk using OCT/OCTA imaging

OCTA serves as a critical tool for assisted diagnosis, progression tracking, and guiding therapeutic decision-making for a wide range of systemic health conditions. Retinal OCTA-derived biomarkers enable non-invasive quantification of microvascular pathology, offering actionable insights to refine clinical decision-making (68). At the same time, Ultra Wide-Field OCTA has been shown to provide reliable performance for detecting neovascularization and intraretinal microvascular abnormalities achieving similar accuracy to fluorescein angiography (69). Traditional assessment methods rely on clinical indicators and patient history, but may suffer from a lack of accuracy and timeliness, especially in areas with relatively scarce resources. Therefore, it is important to explore new, non-invasive assessment methods. Studies have shown that a reduction in the thickness of inner segment/outer segment junction of the retinal pigment epithelium and inner nuclear layer to external limiting membrane is significantly associated with heart failure (55). This study analyzed retinal OCT features using ML models (XGBoost, etc.) and achieved the first non-invasive classification of heart failure subtypes with the best performance (AUC 0.70) (55).

It has been shown that increased CVD risk is inversely related to leukocyte telomere length, whether assessed by traditional biomarkers, CVD risk scores, or our DL heart biological age (BioAge) CVD risk model (56). Shortened leukocyte telomere length serves as an alternative biomarker for increased CVD risk. This model reliably captures this CVD risk biomarker and accurately distinguishes between high and low risk individuals, consistent with known CVD risk trends. Therefore, it can facilitate rapid and accurate screening for CVD risk (56). The ASI-Net model study demonstrates the applicability of AI-enhanced OCTA imaging analysis in the detection of ischemic stroke and its subtype classification (68). Collectively, these advances position OCTA and AI-enhanced retinal analysis as transformative approaches for systemic disease management, bridging critical gaps in early detection, risk stratification, and personalized therapeutic planning across cardiovascular conditions.

### 3.3 AI use multimodal methods to assist in the diagnosis of CVD, predict CVD risk, and stratify patients

AI models use multimodal analysis to identify vascular and retinal abnormalities for the assisted diagnosis of CVD, prediction of CVD risk, and stratification. Compared to single-modal AI models, these models collect more comprehensive information and exhibit higher accuracy. However, the clinical utility of these models remains to be improved. Future research should prioritize large-scale validation of these AI models across different populations and clinical settings to bridge the gap between experimental performance and real-world implementation, ultimately enabling precise CVD prevention strategies. For specific details on each article, please refer to Table 3.

**Table 3:** AI models use multimodal methods to assist in the diagnosis of CVD, predict CVD risk, and stratify patients.

Al algorithm	Country	Dataset	Year	Device used	Disease type	Imaging type	Model performance
DL based Algorithm (70)	UK, China	111,360 individuals	2024	500-MHz Bruker AVANCE III HD NMR, 600-MHz Bruker AVANCE III HD NMR	Predict CVD	CFP	The model combines CFP, metabolomics data, clinical and demographic data, and genetic data to predict CVD with statistical significance (*p* < 0.05).
Multimodal DL model combining CNN and DNN (71)	Korea, UK	14,816 individuals	2023	TRC-SODX Retinal Camera	Predict CVD risk	CFP	The model combines CFP and traditional risk factors, significantly improving the predictive performance of CVD. The model performed well in both internal and external validation.
EfficientNet-B3 (72)	US	8,969 individuals	2025	Not Detailed	Predict CVD risk	CFP	EfficientNet-B3 multimodal model achieves high-precision prediction of CVD risk (AUC-ROC 96.3%) by combining CFP with clinical data, significantly outperforming traditional models such as ResNet-50 and VGG16.
Siamese SE ResNeXt (73)	China	2,903 individuals	2025	Canon CR-2 PLUS AF	Predict CVD risk	CFP	Siamese SE ResNeXt model combined with CFP and clinical data to predict CVD risk AUC-ROC 90.41%.
Multimodal model combining CNN and DNN (74)	UK	30,398 individuals	2018	Topcon 3D OCT-1000 Mark II	Predict CVD risk	CFP	By combining traditional risk factors and retinal features, the model demonstrates high accuracy in predicting the risk of coronary artery disease in HIV-infected individuals (AUC close to 0.99).
Multimodal CNN model (75)	India	112 individuals	2025	ZEISS VISUSCOUT 100 Handheld Fundus Camera	Predict CVD risk	CFP	The accuracy rate of multimodal AI models combining ECG and CFP to predict CVD risk reached 84%.
ECG and CFP model (76)	Spanish	242 individuals	2025	SD-OCT, EDI-OCT	Predict CVD risk	OCT	The AUC for predicting carotid plaque risk using the model was 0.82–0.85.
EfficientNet-B2 network (77)	UK	6,127individuals	2025	Not Detailed	Predict the 10-year MACE risk	CFP	The model effectively predicts the 10-year MACE risk in patients with type 2 diabetes using CFP, with performance comparable to traditional clinical scores (PCE).
UKBiobank-based prognostic models (78)	Europe, UK	95,463 individuals	2022	Topcon 3D-OCT 1000 Mark II	Predicting MI risk	CFP	The model combines CFP, clinical data, and genomic data, achieving performance comparable to or slightly better than the traditional FRS in predicting myocardial infarction risk.
Multimodal model combining CNN and DNN (79)	UK	30,398 individuals	2018	Topcon 3D OCT-1000 Mark II	Predict the 5-year MACE risk	CFP	The model achieved an AUC of 0.70 from retinal fundus images alone, comparable to the AUC of 0.72 for the European SCORE risk calculator.
Multimodal model combining CMR and CFP (80)	UK, US	8,673 individuals	2020	Topcon 3D OCT-1000 Mark II	Predict MI risk	CFP	A multimodal AI model combining CFP, CMR imaging, and demographic data performs comparably to traditional CVD risk models (such as the Framingham score) in predicting MI risk.
Photoreceptor Metabolic Window (81)	UK, China	124,812 individuals	2025	Topcon 3D OCT-1000 Mk II/DRI OCT Triton	Predict MI risk	OCT	A model combining OCT, metabolomics, and clinical data significantly improved the prediction of MI risk.
L1-regularised logistic regression lasso, VAMPIRE 3.1 (82)	UK	3,891 individuals	2019	Not Detailed	StratifyMACE risk in patients with type 2 diabetes	CFP	Multimodal models integrating retinal, genomic, and clinical data can predict MACE risk in patients with type 2 diabetes and effectively distinguish between high-risk and low-risk patients.
VAMPIRE (83)	UK	5,152 individuals	2022	Not detailed	Stratify 10-year MACE risk in patients with type 2 diabetes	CFP	The model effectively predicts and stratifies the 10-year MACE risk of type 2 diabetes patients by combining CFP with genomic data.
Reti-CVD (84)	Republic of Korea	1,106 individuals	2024	Visucam NM/FA	Predict 5-year CVD risk and stratify	CFP	The model predicts and stratifies 5-year CVD risk by combining CFP, clinical data, and other biomarkers. Its predictive accuracy is 0.751, and the risk ratio of the three estimated CVD risk groups is 2.02.
SIVA-DLS (85)	Asia	860 individuals	2023	Canon CR-1 Mark-II Non-mydriatic Digital Retinal Camera	Predict CVD risk and stratification	CFP	Research supports retinal microvascular assessment as a low-cost, noninvasive CVD risk stratification tool.
CLAiR (86)	UK, US	53,145 individuals	2024	Not detailed	Predict 10-year ASCVD risk and stratification	CFP	The model combines CFP and clinical data to achieve stratified 10-year ASCVD risk.
VGG, ResNet (18)	Spanish	152 images	2022	Not detailed	CAC risk stratification in patients with diabetes	CFP	The model integrates CFP and clinical data, with an accuracy rate of 72% for stratifying CAC risk in diabetic patients.
M2AI-CVD (87)	UK, Korea	over 573 individuals	2024	Not detailed	Predict CVD risk	CFP	Combining CFP and clinical data, high-precision (95.89%) CVD risk prediction was achieved.
DXA Model and Retinal Image Model (88)	Qatar	1,805 images	2022	Topcon TRC-NW6S retinal camera	Diagnose CVD	CFP	A multimodal DL model combining CFP and DXA data achieved an accuracy rate of 78.3% in diagnosing CVD.

#### 3.3.1 AI predicts CVD risk using multimodal

As research on AI models predicting CVD through retinal imaging continues to deepen, more and more models are combining clinical data, genomic data, metabolomic data, and other information to comprehensively predict CVD risk and improve model efficiency (89, 90). The study used DL models and metabolomics technology to reveal the molecular link between retinal aging and CVD, establishing a novel biomarker called the metabolomic signature of retinal aging (MSRA) (70). It also verified that MSRA has statistical significance in predicting CVD (*p* < 0.05) (70). A multimodal DL model combining CNN and deep neural network (DNN) significantly improved the predictive performance of CVD by combining CFP and traditional risk factors (such as age, blood pressure, cholesterol, etc.) (71). The model performed well in both internal and external validation and was able to identify high-risk patients for future CVD events (71). The efficientnet-base model variant 3 (EfficientNet-B3) multimodal model achieves high-precision prediction of CVD risk (AUC-ROC 96.3%) by integrating CFP with clinical data, significantly outperforming traditional models such as residual neural network with 50 layers (ResNet-50) and VGG16 (72). Its core advantage lies in combining clinical data to overcome the limitations of single data sources, thereby enhancing the comprehensiveness of predictions (72). On the other hand, it uses gradient-weighted class activation mapping (Grad-CAM) to generate heatmaps, visualizing the model's focus on critical retinal regions (such as areas with vascular abnormalities), thereby enhancing the model's credibility (72). A study achieved high-precision prediction of CVD risk based on CFP (AUC-ROC 90.41%) through the fusion of multimodal data with the siamese squeeze-and-excitation resnext (Siamese SE-ResNeXt) model (73). The dataset used for this model is “China-Fundus-Carotid Intima-Media Thickness (CIMT) dataset”. This dataset is an integration of retinal and carotid intima-media thickness data to provide a reference for the creation of new datasets in the future. Moreover, it is also an important resource for the development and validation of AI-based early CVD screening models using retinal imaging (73). Research has found that by combining traditional risk factors and retinal characteristics, AI models demonstrate high accuracy in predicting the risk of coronary artery disease in human immunodeficiency virus (HIV)-infected individuals (AUC close to 0.99) (74).

A study used a multimodal AI model [Electrocardiogram (ECG) + CFP] to fuse spatiotemporal features using fast fourier transform + earth mover's distance, achieving an 84% accuracy rate in predicting CVD risk, with a particular strength in identifying early microvascular lesions (75). A study combining ML and OCT technology confirmed that reduced choroidal thickness in patients with type 1 diabetes is significantly associated with carotid plaque (76). Its AUC for predicting carotid plaque was 0.82–0.85 (76). Research has found that DL models based on efficientnet-base model variant 2 (EfficientNet-B2) can efficiently predict the 10-year major adverse cardiovascular events (MACE) risk of type 2 diabetes patients through CFP, with performance comparable to traditional clinical scores (PCE) (77).

A new AI model combines retinal imaging, clinical data, and genomic data to achieve performance comparable to or slightly better than the traditional FRS in predicting the risk of MI (78). The combination of CFP and DL technology has enabled 5-year risk prediction for MACE, with an AUC of 0.7 (79). The AUC is comparable to the AUC of 0.72 for the European SCORE risk calculator (79). Multimodal AI models, combining CFP, cardiac magnetic resonance (CMR) images, and demographic data, perform as well as traditional CVD risk models (such as the Framingham score) in predicting MI risk, while being less expensive and more accessible (80). The AI-driven photoreceptor metabolic window model integrates OCT, metabolomics, and DL. This model significantly improves the prediction of MI risk and reveals the metabolic basis of the relationship between photoreceptor layer thickness and the risk of multisystem diseases (81).

In summary, currently, there are relatively more studies combining clinical data and genomics with AI models based on CFP, and their predictive performance has improved to a certain extent. However, there are fewer multi-modal AI models based on OCT and OCTA, but their performance is better than that of single-modal models. Multi-modal models combining OCT, CFP, OCTA and other clinical data have great potential in predicting CVD risk and are worth further research. This shows that in the future we can try to integrate OCT, OCTA, CFP and other prediction methods. This will not only improve the prediction rate of CVD risk, but also refine the risk stratification more. This helps with early management.

#### 3.3.2 AI stratifies CVD risk using multimodal

Early detection and risk stratification of CVD are crucial for prevention and treatment. The accuracy of predicting CVD risk and stratification using single imaging or assisted examinations needs to be improved, while multimodal fusion technology can significantly improve predictive accuracy. A multimodal AI model integrating retinal, genomic, and clinical data can predict the risk of MACE in patients with type 2 diabetes and effectively distinguish between high-risk and low-risk patients (82). Compared with DL models, the features selected by Lasso regression in this model have clear clinical significance, enhancing the interpretability of the model (82). By analyzing vascular parameters in CFP and combining them with genomic data, the AI model vascular mapping and perfusion imaging reconstruction (VAMPIRE) can effectively predict and stratify the 10-year MACE risk in patients with type 2 diabetes (83). The model's AUC for predicting MACE is 0.663, comparable to the traditional PCE risk score (AUC 0.658) (83). When combined with retinal parameters and a polygenic risk score, the AUC improves to 0.686, significantly outperforming the PCE risk score (83).

The AI algorithm based on DL (Reti-CVD) combines CFP, clinical data, and other biomarkers to predict 5-year CVD risk and stratify it. Its predictive accuracy is 0.751, and the risk ratio for the estimated three CVD risk groups (low, medium, and high risk) is 2.02 (84). This model is equivalent to CAC scoring, but is less costly and easier to operate (84). The SIVA-DLS model combines retinal vascular parameters and clinical data to achieve an AUC of 0.760 for predicting CVD risk (compared to an AUC of 0.720 for traditional risk factors) (85). The study also supports retinal microvascular assessment as a low-cost, non-invasive CVD risk stratification tool, particularly suitable for resource-limited areas (85). The CLAiR model uses CFP and limited demographic data to predict and stratify 10-year atherosclerotic CVD risk (86). The AUROC for predicting atherosclerotic CVD (ASCVD) risk is 0.89–0.9. Its risk stratification ability is consistent with traditional ASCVD risk assessment (86).

In summary, multimodal AI models that integrate retinal imaging, genomics, and clinical data demonstrate superior predictive performance for CVD risk stratification compared to traditional methods like PCE risk score, while also offering cost-efficiency and scalability. These advancements highlight the potential of AI-driven, interpretable risk assessment tools to enhance early CVD detection and personalized prevention strategies in high-risk populations, such as type 2 diabetes patients.

#### 3.3.3 AI assisted diagnoses CVD using multimodal

The AI model combining VGG16 and transfer learning integrates CFP and clinical data to determine CAC in diabetic patients and stratify CAC risk (with an accuracy rate of 72%) (18). It also innovatively combines clinical data to optimize predictions (with an accuracy rate of 91%) (18). The M2AI-CVD system combines CFP with clinical data and improves model performance through entropy-optimized segmentation and genetic algorithm feature selection (87). It achieves high-precision (95.89%) CVD risk prediction (87). This provides a promising solution for the accurate and early detection of CVD (87). This study is the first to propose a multimodal DL model combining CFP and dual-energy x-ray absorptiometry (DXA) data for non-invasive CVD assisted diagnosis, with an accuracy rate of 78.3% (88). Despite data limitations and generalization challenges, its non-invasive, efficient, and interpretable nature offers new insights for early CVD screening (88). In the future, by expanding the dataset and conducting clinical validation, it is expected to become a powerful tool for rapid CVD screening in primary care.

## 4 Discussion

### 4.1 Current status of AI using retinal imaging to predict CVD

In recent years, there has been an increasing number of applications based on AI to automate imaging processing (91). Significant progress has also been made in the application of AI to medical imaging analysis, especially in predicting and evaluating CVD through retinal imaging (92). Research shows that AI-based identification of retinal biomarkers has great potential in predicting CVD (47, 93). A study found that retinal blood vessels may be a potential biomarker for coronary artery atherosclerosis (94). Through DL, convolutional neural networks, and other methods, AI technology is able to extract biomarkers associated with CVD from retinal imaging. This provides new tools for early assisted diagnosis, risk assessment and prognosis prediction of CVD. Currently, there are many AI models based on CFP, but few based on OCT and OCTA. Multimodal models typically combine retinal imaging with clinical data, demographic data, metabolomics, genomics, ECG, CMR, DXA, and other information. Overall, multimodal models can collect more comprehensive information and have better predictive performance, making them an important direction for future research.

With the increasing application of AI in the medical field, CVD risk prediction technology based on CFP has developed rapidly. Traditional CFP analysis relies on the experience of doctors, which is subjective and time-consuming. Through DL models, AI technology can quickly and accurately identify subtle changes in CFP (95, 96). Research indicates that CFP contains information about future CVD risk, and retinal microvascular features (such as ischemic perivascular lesions, density, and vessel diameter) can serve as assessment criteria for systemic diseases (97). For example, AI models can predict the risk of CVD such as hypertension and atherosclerosis by analyzing retinal vessel diameter, curvature, and branching angle (26, 39). The U-Net57 model revealed the association between the retina and congestive heart failure by analyzing microvascular density and fractal dimension, while the model based on the Inception-v3 architecture achieved an AUC of 83.2% in predicting high coronary artery calcium scores (CACS > 100), outperforming single clinical parameters. Additionally, multi-feature fusion models (e.g., Hybrid Inception V3-VGG16) achieve accuracy as high as 99.5%, combining efficiency and non-invasiveness, making them particularly suitable for early screening in resource-limited regions. In terms of risk stratification, AI models such as rpCVD (AUC = 0.672) and Reti-CVD (10-year CVD risk of 13.1% in the high-risk group) achieved stratification capabilities comparable to WHO scores and QRISK3 through CFP, and could even identify high-risk populations missed by traditional tools. The DL-FAS model optimizes decision support for moderate-risk populations by quantifying retinal atherosclerosis features (AUROC = 0.713) (41). While existing models generally exhibit high accuracy, most rely on single-center, small-sample data, limiting their generalization capabilities. Additionally, AI has demonstrated potential in exploratory diagnostics for specific CVD subtypes (e.g., carotid atherosclerosis, peripheral artery disease), but its clinical utility remains to be validated.

Multimodal AI models based on retinal imaging have seen rapid development in the field of CVD risk prediction. The core breakthrough lies in the integration of multimodal data and the improvement of model interpretability. Research shows that by integrating clinical data (such as age and blood pressure), genomics (polygenic risk scores), and metabolomics (such as retinal aging metabolic markers MSRA), the model performance significantly outperforms traditional risk assessment tools. For example, the EfficientNet-B3 model (AUC 96.3%) and the Siamese SE-ResNeXt model (AUC 90.41%) outperform ResNet-50 and the Framingham score, respectively, by combining CFP with clinical features.

Notably, the multimodal strategy demonstrated unique advantages: after integrating CFP vascular parameters with genomic data, the VAMPIRE model improved MACE prediction AUC from 0.663 to 0.686. While the photoreceptor metabolic window model, which combines OCT, CFP, and metabolomics, revealed the metabolic mechanisms linking retinal thickness to the risk of multisystem diseases. In terms of risk stratification, the Reti-CVD model achieved a risk ratio of 2.02 across low, medium, and high-risk groups through 5-year risk prediction (accuracy of 0.751), with performance comparable to CACS but at lower cost; the CLAiR model achieved an AUROC of 0.89–0.9 using CFP and basic demographic data, validating the potential of retinal imaging to replace complex examinations. Current challenges include limited research on OCT/OCTA multimodal models and reliance on single-center data (e.g., the China-Fundus-CIMT dataset) for some models.

### 4.2 Comparison of models based on different retinal imaging

Current evidence suggests that AI models using CFP generally achieve higher predictive accuracy for CVD risk than those based on OCT or OCTA. CFP-based models also benefit from larger training and validation datasets, enhancing their reliability. Nonetheless, OCT/OCTA-based models show high accuracy in predicting MI risk. Multimodal approaches, which integrate demographic data, retinal imaging, and other clinical variables, can effectively predict CVD, MI, and MACE, with some models performing comparably to the PCE risk score and even surpassing the FRS.

Although relatively few AI models are designed specifically for CVD risk stratification, several show promising performance. For example, the CFP-based rpCVD model achieved 63.4% agreement with WHO risk categories, and its use of a large dataset enhances credibility. Similarly, multimodal models can stratify risks for MACE, CVD, and ASCVD with accuracy comparable to or exceeding PCE scores. However, these models often rely on datasets with selection bias, underscoring the need for improved internal and external validation. As AI technology advances, a growing number of retinal imaging-based models are being developed for CVD diagnosis. CFP-based approaches have shown utility in diagnosing carotid atherosclerosis, CAC, and PAD, often outperforming single clinical parameters such as age or blood glucose levels. In contrast, multimodal models are currently limited to assisting in the diagnosis of CAC and general CVD. Moreover, their constrained dataset availability results in lower accuracy, reliability, and generalizability compared to CFP-based models.

In summary, AI models based on CFP are more extensively developed and typically utilize larger datasets than those employing other imaging modalities. Several CFP-based models have undergone both internal and external validation, demonstrating stable accuracy and robust generalizability. In contrast, the use of OCT and OCTA for CVD risk prediction and stratification represents an emerging field, with relatively few models available and their diagnostic utility still under investigation. Recent research has increasingly focused on multimodal AI approaches, which integrate retinal imaging with demographic and clinical data to achieve superior predictive and stratification performance. These models are likely to become a major focus of future research due to their enhanced capabilities. However, their development is constrained by the need for large, complex datasets and advanced technical infrastructure, making them currently less suitable for resource-limited settings. In comparison, CFP-based AI models—compatible with portable fundus cameras—offer greater scalability for large-scale screening in underserved populations. A detailed comparison of AI models based on CFP, OCT, OCTA, and multimodal data is provided in Table 4.

**Table 4:** AI predicts, stratifies, and assists in diagnosing CVD through various retinal imaging.

Model	Type	Disease	Dataset Size	Results	Performance	Limitations
CFP-based model	Predict Risk	CACS	20,130 individuals	The model AUROC reached 0.832	Outperform single clinical parameters	The population is homogeneous and lacks multi-ethnic validation; it has not met clinical deployment standards (typically requiring >90%); the mechanism remains unclear.
		ICVD	412,827 individuals	The model's AUC value ranges from 0.85 to 0.971	Superior to the traditional questionnaire + blood test model	The CNN decision-making process is difficult to explain; the BRAVE cohort is older and has a higher proportion of women.
		CVD	1,000–210,494 images	The model's AUC value ranges from 0.779 to 0.995	Some models outperform traditional model, and even surpass or match expert performance.	Some models have small sample sizes; samples come from a single source; external validation is insufficient or lacking; quality depends on retinal imaging; reliability needs improvement.
	Risk Stratification	CVD	Over 27,595 individuals	The rpCVD model showed 63.4% agreement with WHO risk scores across risk categories	Some models demonstrate comparable risk stratification performance to CT-determined CAC scores, and achieve finer stratification within the critical risk group.	Sample homogeneity; sample selection bias; training data bias; inconsistent imaging equipment.
	Assisted Diagnosis	Carotid atherosclerosis	37,523 individuals	AUROC: 0.713. AUPRC: 0.569. Accuracy: 0.583, Sensitivity: 0.891, Specificity: 0.404.	Compared to the simpler FRS model, incorporating DL-FAS improves concordance by 0.0266.	The specificity for predicting carotid atherosclerosis is only 0.404, which may lead to a higher number of false positives.
		CAC	216,152 images	The model predicted an AUC of 0.742 for CAC.	Predictive accuracy surpasses that of single clinical parameters such as age and blood glucose levels.	Training data bias: The development set originates from health screening centers, potentially excluding high-risk or special populations. Event definition bias: Some datasets utilize administrative data to define CVD events, which may introduce misclassification.
		PAD	135 individuals	The AUC of this model for diagnosing PAD is 0.89.	Early diagnostic potential: Demonstrates significant effectiveness in detecting asymptomatic PAD (Fontaine Stage I)	Sample size is extremely limited, with a single source; no independent cohort validation has been conducted; high-resolution imaging impose demanding requirements on AI models.
OCT/OCTA-based model	Predict Risk	Neurocardiovascular	491 individuals	AUC of 0.68–0.98 and an accuracy of 85%.	SS OCT-A delivers micrometer-level retinal microvascular imaging, surpassing traditional fundus photography.	The sample size is limited, restricting its generalizability. Device dependency exists, as it is based on specific equipment and has not been validated on other devices. Data imbalance is present, with uneven gender distribution between the medium-to-high risk group and the low-risk group.
		CVD	144 individuals	The model's accuracy in predicting CVD risk ranges from 75.64% to 96.53%.	Superior to traditional risk models. Utilizing supervised learning, the model offers greater transparency and higher interpretability.	Small sample size. Data labeling may result in loss of continuous variable information. Device dependency on specific OCT-A and fundus cameras. Cross-sectional study, not a prospective study. Not externally validated.
		MI and heart failure	1,640,612 images	The model predicted AUROC values of 0.737 and 0.794 for MI and heart failure.	The RETFound model outperforms other comparative models	Large sample size, multicenter study, but no comprehensive analysis; external validation results were not clearly reported;
		congestive heart failure	247 individuals	The AUC for predicting congestive heart failure risk was 0.61.	The higher the resolution of the imaging, the higher the prediction accuracy.	Small sample size, single-center study. Selection bias, primarily involving cardiovascular inpatients with a high proportion of males. Clinical data not integrated.
		coronary artery disease	120 individuals	The accuracy of the model is up to 86%	Significantly higher than traditional logistic regression models (78.7%).	Small sample size; Equipment variability, with no clarification on the uniformity of OCTA devices; Population limitations; Lack of external validation using independent cohorts.
	Risk Stratification	heart failure subtypes	2,824 individuals	The AUC value of this model is 0.70.	Some models achieved an AUC >0.68 in the CHF/UHF classification, outperforming traditional clinical indicators.	Data limitations. Subtype classification variations resulted in poor predictive performance for LVHF (AUC 0.61). The cross-sectional design precludes establishing a causal relationship between retinal changes and heart failure.
		CVD	33,370 individuals	This model can accurately distinguish between high-risk and low-risk individuals.	This model aligns with known trends in CVD risk.	Cross-sectional studies cannot establish causality; The sample primarily consists of European whites; The model's performance on other datasets remains unverified.
Multimodal model	Predict Risk	CVD	112–111,360 individuals	The AUC value of models is 0.84–0.99.	Significantly outperforms traditional risk prediction models (with an AUC improvement of approximately 0.2–0.3).	Data limitations prevent capture of dynamic changes. The study population is primarily based on European individuals. No assessment of NMR equipment uniformity was conducted.
		MACE	6,127individuals	The model's AUC is 0.697.	Equivalent to the commonly used PCE risk score in clinical practice.	Population limitations: Primarily white individuals aged 60–75. Applicable only to low-risk populations. Technical limitations: Low-resolution imaging may result in loss of detail.
		MI	8,673–124,812 individuals	AUC ranges from 0.737 to 0.8.	Some models perform close to or even better than FRS scores.	Equipment Variation: Different OCT devices were used for database collection, but analysis standards were consistent. Uncontrolled Confounding Factors: Such as refractive errors, macular degeneration, etc.
	Risk Stratification	MACE	3,891–5,152 individuals	Effectively distinguish between high-risk and low-risk patients.	Some models outperform the PCE risk score.	The study was limited to patients with type 2 diabetes; external validation was lacking; parameters were limited; and semi-automated measurements restricted processing efficiency.
		CVD	860–1,106 individuals	AUC ranges from 0.72 to 0.751.	Low-cost and non-invasive, particularly suitable for resource-limited areas.	Sample size is limited; racial diversity is lacking; algorithm details remain undisclosed; longer follow-up is required to validate risk stratification.
		ASCVD	53,145 individuals	Internal validation AUROC = 0.89; External validation AUROC = 0.90	Comparable to PCE in predicting ASCVD events.	Predictive performance among Black populations exhibits small but statistically significant differences compared to other racial groups; the real-world data used to train deep learning models likely lacks reliability.
	Assisted Diagnosis	CAC	152 images	with an accuracy rate of 72%.	There are clinical diagnostic models and large-scale screening models, which are widely applied.	Small sample size limitations; Equipment non-standardization: Retinal camera model not specified; Population limitations.
		CVD	over 573 individuals	AUC ranged from 78.3% to 95.89%.	By integrating clinical data with DXA scan results, predictive capabilities have been enhanced.	Data limitations; AUC not specified; insufficient clinical validation; high computational complexity.

### 4.3 Clinical utility of AI models

AI models relying exclusively on CFP demonstrate high predictive accuracy for CVD, with performance comparable to or exceeding that of established risk assessment tools. For example, the CNN-based Singapore I Vessel Assessment–Deep Learning System (SIVA-DLS) model showed strong agreement with expert evaluations, achieving an intraclass correlation coefficient of 0.82–0.95 for CVD risk factor prediction (26). Similarly, the RetiCAC model yielded an AUC of 0.742 for predicting CAC and significantly improved the predictive capacity of the PCE score in intermediate- and borderline-risk groups (39). The rpCVD model achieved an AUC of 0.672, which is comparable to the WHO CVD risk score (AUC = 0.693), with 63.4% of participants showing consistent risk stratification (low/moderate/high) between the two approaches (37). Additionally, incorporating the DL–based feature augmentation strategy (DL-FAS) model alongside the FRS improved concordance by 0.0266 compared to using FRS alone (41).

Current multimodal models are frequently benchmarked against established risk scores such as the PCE and FRS. Studies indicate that the EfficientNet-B2 model achieved an AUC of 0.697 for predicting MACE, comparable to the PCE score; when integrated with a polygenic risk score for coronary artery disease and PCE, performance improved to an AUC of 0.728 (77). Similarly, the VAMPIRE model yielded an AUC of 0.663 for MACE prediction—on par with PCE (AUC 0.658)—and reached 0.686 when augmented with retinal parameters and genetic risk data, significantly surpassing PCE alone (83). The CLAiR model also demonstrated predictive capability for ASCVD events comparable to that of PCE (86). A multimodal achieved an AUC of 0.7 for MACE prediction, comparable to the European SCORE risk calculator (79). In external validation, a model integrating CFP, CMR imaging, and demographic data attained an AUC of 0.70 for MI prediction, approaching the performance of FRS (80). The QUARTZ model has also been reported to perform comparably or slightly better than FRS in predicting MI (78). Overall, multimodal models generally exhibit stronger predictive ability for CVD risk than unimodal approaches, with many matching or exceeding conventional risk tools. Integrating monomodal AI models with PCE scores can further enhance risk assessment accuracy, underscoring a key advantage of AI-enhanced retinal imaging in CVD prediction.

AI models based on retinal imaging offer a promising approach for large-scale cardiovascular screening due to their non-invasive nature, operational efficiency, and strong scalability (39, 65). These attributes make such models particularly suitable for regions with limited medical resources (75). The screening process requires only retinal imaging, eliminating the need for blood draws or complex examinations, which enhances patient acceptability (87). The procedure is highly efficient, with a median imaging time of approximately one minute and 47 s per eye and a 93.9% image quality pass rate, while certain models achieve predictive accuracies as high as 99.5% (29, 36, 37). The workflow—from image preprocessing to risk classification—is fully automated, reducing reliance on operator skill and increasing reproducibility (32). In healthcare systems where fundus examination is already part of routine checkups, DL models such as DL-FAS can be applied directly to existing images without incurring additional examination costs (41). Architectures such as EfficientNet-B3, which have fewer parameters and lower computational demands, are especially suitable for low-resource clinical environments (72). The widespread availability of fundus cameras in primary care and ophthalmology clinics further supports the scalability of these tools (78, 84). Nevertheless, the clinical translation of retinal imaging-based AI models continues to face challenges. These include limited and often imbalanced training datasets, the lack of unified image quality standards, limited model interpretability, and insufficient validation of performance stability—all of which hinder broad implementation.

### 4.4 Limitations and shortcomings

Despite the increasing number of AI models being developed and validated, current research still faces many challenges.

#### 4.4.1 Population limitations and insufficient generalization ability

Existing models are primarily developed for specific populations (e.g., diabetic patients), and their generalization ability across populations and races has not been sufficiently validated. Moreover, the diversity and quality of datasets vary significantly, affecting the stability of model performance.

#### 4.4.2 Technical limitations

On the one hand, microvascular changes observed through retinal imaging may lack specificity for certain diseases. On the other hand, differences in imaging quality and parameters among different retinal imaging devices lead to inconsistent model performance across devices. Additionally, the lack of a unified database of normal values and imaging acquisition protocols hinders the comparability of results. Furthermore, motion artifacts or signal attenuation may also interfere with the accuracy of analysis.

#### 4.4.3 Model interpretability and public acceptability

Due to the lack of clear AI decision-making processes and quantitative metrics or standards, AI is often viewed as a “black box” (98). This can lead to skepticism among doctors and patients regarding the results. Additionally, the data-driven nature of the model makes it susceptible to biases in the training data. This further exacerbates public distrust of AI.

### 4.5 Future prospects

#### 4.5.1 Deepening multimodal fusion technology

Compared with single-modal models, multimodal models have better predictive performance. On the one hand, they can integrate technologies for identifying various types of retinal imaging. The synergistic application of multiple retinal imaging technologies, such as CFP, OCT, and OCTA, can be explored and combined with computer vision technology and self-supervised learning models (32, 99) to improve predictive accuracy. On the other hand, cross-modal data fusion can be conducted. By integrating genomic and metabolomic data (e.g., the photoreceptor metabolic window model), the specificity and clinical value of the model can be enhanced. Additionally, the model can be deployed in clinical settings for validation. Referencing models combining ECG with retinal imaging (75) and studies combining OCTA parameters with carotid artery stenosis (100), the validation of multimodal models in real clinical scenarios can be advanced.

#### 4.5.2 Improving model interpretability

The lack of interpretability in AI models significantly impacts the trust doctors and patients have in them. On the one hand, Visualization techniques should be used as much as possible to identify retinal imaging. For example, gradient-weighted class activation mapping (Grad-CAM) heatmaps (28, 32, 49) and shapley additive explanations (SHAP) models (101) can be used to visually demonstrate the key retinal regions the model focuses on (such as blood vessels, the macula, and the optic disc). Analyze the anatomical structures relied upon by the model using attention weights to enhance clinical credibility (80). On the other hand, mathematical modeling methods can be explored to improve interpretability. Develop interpretable algorithms such as adaptive elliptical templates (102) to maintain robustness under conditions of abundant lesions or low contrast, thereby addressing the “black box” challenge of AI. Research suggests that explainable AI (XAI) technology can reveal black-box ML models, enhancing model credibility and reliability (103). One study incorporated SHAP analysis into AI models to achieve better model understanding (101, 104). Future AI models may incorporate XAI technology and SHAP analysis to improve model credibility (101, 103, 104). Additionally, the model combines multi-scale feature extraction and fusion techniques with a dual attention mechanism, which promotes the extraction of multi-scale vascular features and may help improve the model's interpretability (105). In addition, a pilot study evaluating HbA1c demonstrated the potential and considerations required to develop reliable AI in the oculomics pilot, which contributes to the transparency of AI models (106).

#### 4.5.3 Standardized database construction and generalization capability optimization

Current AI models for CVD screening are predominantly trained on limited, single-center datasets, which restricts their generalizability and clinical applicability. There is a pressing need to develop large-scale, multi-center, and multi-ethnic datasets to enhance model robustness and performance across diverse populations. For instance, the Retinal OCT Angiography and Cardiovascular Status (RASTA) dataset includes retinal microvascular imaging from 499 patients, featuring 814 vascular cuboids and 2,005 facial images, and represents the only publicly available resource with imaging data from both healthy individuals and high-risk CVD populations (107). This dataset is expected to facilitate the development of universal screening models using OCT-A imaging (107). Similarly, the mBRSET dataset—the first publicly available diabetic retinopathy resource—contains 5,164 retinal images from 1,291 ethnically diverse patients, all acquired using handheld cameras, thereby addressing data scarcity in low- and middle-income settings (108). A related study outlines steps for constructing a large-scale online retinal imaging database in India, offering a replicable framework for cost-effective, AI-based diagnostic tool development (109).

To improve model generalizability, future efforts should prioritize standardized, multi-institutional datasets that incorporate varied imaging devices (e.g., portable cameras) and population characteristics. External validation across diverse datasets—such as the UK Biobank and Eye Picture Archive Communication System (PACS), as performed for the CLAiR model—is also essential. One study reported a DL model capable of predicting glaucoma progression with varying accuracy across ethnic groups (76.9% in Caucasians, 14.6% in African Americans, and 8.5% in Asians), highlighting the importance of ethnically balanced training data (22). Insights from such studies can inform the design of more generalizable AI systems (13, 22). Finally, establishing unified data annotation standards will be critical to supporting reproducible and scalable model development.

#### 4.5.4 Clinical application translation strategies

Due to the non-invasive, efficient, and convenient nature of retinal imaging, they are particularly suitable for primary care settings and resource-limited regions. Therefore, future efforts could focus on designing portable devices to enhance clinical practicality. A study comparing automated and semi-automated methods for measuring retinal microvascular biomarkers found good correlation between the two approaches in assessing vascular complexity and vessel diameter measurements, with consistent clinical relevance (110). However, the automated model exhibited a higher rejection rate within the dataset (110). Before transitioning from semi-automated to automated algorithms in retinal microvascular biomarker analysis, further comparative research is warranted (110). Additionally, dynamic risk assessment models could be developed to integrate long-term follow-up data on retinal changes and CVD progression for personalized management.

## 5 Conclusion

In recent years, AI technology based on retinal imaging has made significant breakthroughs in the fields of assisted diagnosis and CVD risk assessment. Single-modality models based on CFP/OCT/OCTA have achieved high-precision predictions and support risk stratification. Multimodal models that integrate genomic, metabolomic, and ECG data have significantly improved performance. Grad-CAM heatmaps and SHAP analysis are gradually unraveling the “black box” of AI, enhancing clinical credibility. However, some issues remain, such as models heavily relying on single-center data, insufficient validation across populations/devices, and room for improvement in generalization. Differences in device parameters and imaging artifacts affect result comparability, and there is a lack of a unified database. In the future, we can draw on the experience of the Indian retinal database and mobile brazilian retinal dataset to build multi-center, multi-ethnic standardized datasets. Additionally, we can develop dynamic risk assessment models that integrate long-term retinal changes with CVD progression. We can also develop and promote portable devices, particularly for resource-limited regions. Retinal imaging AI holds promise for advancing early CVD prevention and control systems, but interdisciplinary collaboration is needed to address issues of generalizability, standardization, and interpretability, thereby achieving a transition from “high-precision prediction” to “high clinical value”.
